# Diurnal Cortisol Rhythm Is Associated With Adverse Cardiac Events and Mortality in Coronary Artery Bypass Patients

**DOI:** 10.1210/jc.2015-2617

**Published:** 2015-08-25

**Authors:** Amy Ronaldson, Tara Kidd, Lydia Poole, Elizabeth Leigh, Marjan Jahangiri, Andrew Steptoe

**Affiliations:** Department of Epidemiology and Public Health (A.R., T.K., L.P., E.L., A.S.), University College London, London WC1E 6BT, United Kingdom; and Department of Cardiac Surgery (M.J.), St. George's Hospital, University of London, London SW17 0QT, United Kingdom

## Abstract

**Purpose:**

There is growing evidence that the hypothalamic-pituitary-adrenal axis plays a role in the progression of cardiovascular disease. We examined the relationship between diurnal cortisol rhythm and adverse events in patients undergoing coronary artery bypass graft (CABG) surgery. We hypothesized that a flatter presurgical diurnal cortisol slope would be associated with higher rates of adverse cardiac events and death in the years following the CABG procedure.

**Methods:**

Repeated measures of saliva were taken over the day from 250 CABG patients 1 month before surgery to assess diurnal cortisol slope and overall output (area under the curve). Long-term clinical outcomes were occurrence of a major adverse cardiac event (MACE) and death, and were collected up to 2.68 (SD = 0.40) years after surgery. Cox proportional hazard models were used to determine relationships between presurgical cortisol and clinical outcomes. EuroSCORE, chronic illness burden, and whether or not the patient had undergone cardiopulmonary bypass were included as covariates in the models.

**Results:**

Diurnal cortisol slope predicted the occurrence of MACE or death after surgery (hazard ratio = 0.73; 95% confidence interval = 0.56–0.96; *P* = .023). Patients with a steeper slope were at reduced risk of adverse outcomes. This association was driven by changes in both waking and evening cortisol levels.

**Conclusion:**

These results provide evidence for a link between diurnal cortisol rhythm and recovery after CABG. Measuring diurnal cortisol slope before surgery may help to identify those patients at risk of adverse outcomes in the years after the procedure.

There is growing evidence that the hypothalamic-pituitary-adrenal (HPA) axis plays a role in the progression of cardiovascular disease (CVD). The HPA axis is a major component of the neuroendocrine system that controls the stress response and a number of important bodily functions. Cortisol is the end product of the HPA axis and influences a number of factors relevant to the etiology of CVD. There is growing evidence that elevated cortisol levels are associated with adiposity, low-density lipoprotein cholesterol levels, endothelial dysfunction, increases in blood pressure, and hemostasis (1). Cortisol also plays a regulatory role in the complex relationship between inflammation and vascular disease (2). Heightened cortisol levels have been associated with subclinical atherosclerosis of the carotid arteries (3) and coronary artery calcification (4). Furthermore, rates of CVD are high in people with Cushing's syndrome—a disease defined by hypercortisolism (5). Elevated 24-hour urinary cortisol has been found to predict cardiovascular death in older people both with and without CVD (6). However, the role of cortisol in patients with advanced CVD is less clear. Higher serum cortisol levels have been found to predict both mortality risk and risk of future cardiac events in chronic heart failure (7) and ischemic stroke (8). However, results from studies of cortisol in acute coronary syndrome have been less consistent (9, 10).

One difficulty in interpreting this evidence is that cortisol is typically measured with a single serum sample. Cortisol shows marked diurnal patterning, and levels vary substantially across the day. Cortisol is at high levels on waking, followed by a rise that reaches a peak approximately 30 minutes after waking. This is referred to as the cortisol awakening response. There is then a subsequent decline across the day, with cortisol reaching its lowest point at around midnight. It is possible that differences in the point during the diurnal cycle at which single serum samples are obtained account for inconsistencies in associations with CVD. Sampling cortisol several times across the day allows for measurement of the diurnal cortisol profile and a more in depth investigation of the associations between cortisol and clinical endpoints. Dysregulation of the HPA axis can result in a reduction in the amplitude of the diurnal pattern or a flatter slope across the day (11). A flatter cortisol slope across the day has been associated with higher levels of coronary artery calcification (11) and increased cardiovascular mortality in nonclinical populations (12).

There is a paucity of studies examining the effects of variations in diurnal cortisol rhythms on future cardiac events and mortality in patients with established CVD. We therefore sought to examine the relationship between presurgical diurnal cortisol and clinical outcomes in patients undergoing coronary artery bypass graft (CABG) surgery. We hypothesized that a flatter diurnal cortisol slope before surgery would be associated with higher rates of future cardiac events and mortality in the years after CABG.

## Subjects and Methods

### Participants

The data we used in this analysis were collected as part of the Adjustment and Recovery after Cardiac Surgery (ARCS) study involving patients undergoing elective CABG surgery or CABG plus valve replacement to participate. CABG surgery in a single center (13) included both on-pump and off-pump procedures. All procedures were carried out with written informed consent of the participants. Ethical approval was obtained from the National Research Ethics Service.

Participants were 262 prospective CABG patients who were recruited from a presurgical assessment clinic at St. George's Hospital, London. Eligible participants had to be at least 18 years of age and had to be able to complete questionnaires in English. Long-term recovery outcomes were collected from electronic and paper patient records on average 2.68 (SD = 0.40) years after surgery. We carried out analyses on 250 patients with complete data on clinical outcomes and cortisol slope. There were no significant associations between the use of steroid medications and cortisol output, outcome variables, or covariates (all *P* > .05). Therefore, patients taking steroid medications (n = 8) were included in the analyses.

There were no significant differences between patients included in and excluded from the analyses in terms of age, sex, the occurrence of major adverse cardiac events (MACEs), chronic disease burden, or whether or not the person had on-pump surgery. However, EuroSCORE was higher in the 12 patients without cortisol follow-up data (*F* (2, 345) = 5.233; *P* = .006) indicating poorer prognosis on average.

### Measures

#### Diurnal salivary cortisol

At the presurgical assessment clinic, participants received a saliva collection kit and were given instructions for collection at home. The kit included seven prelabeled “salivette” collection tubes (Sarstedt) and a cortisol diary. The cortisol diary contained instructions on how and when to give samples. These diaries were also used to record information on factors likely to introduce variation in cortisol samples such as mood, exercise, and daily stressors. Participants provided seven saliva samples over the course of a weekday: on waking, 30 minutes after waking (30+), 10 am, noon, 4 pm, 8 pm, and bedtime. Participants stored their samples in the refrigerator before returning them to the clinic. The samples were obtained on average 30.6 days (SD = 36.9) before surgery. Cortisol levels were assessed from saliva using a time-resolved immunoassay with fluorescence detection at the University of Dresden, Germany.

We computed total cortisol output over the day by calculating the cortisol area under the curve (AUC) with respect to ground (14). The cortisol slope was calculated in nanomoles per liter per hour (nmol/L/h) by regressing cortisol on sample collection time, with 30+ excluded; higher values indicate a steeper decrease in cortisol over the day. Waking and evening (the average of 8 pm and bedtime) values were also calculated.

#### Long-term clinical outcome

Long-term clinical outcomes were occurrence of a MACE and death (all-cause mortality) and were collected up to 2.68 years after surgery. Postoperative MACE included admissions for myocardial infarction, unstable angina, stroke, and/or heart failure. Occurrence of MACE was treated as a binary variable where either no MACE occurred or at least one MACE occurred. Mortality data were gathered by reviewing in-hospital electronic and paper patient records.

### Covariates: clinical, sociodemographic, and psychosocial factors

Cardiovascular history and clinical factors during admission and management were obtained from clinical notes. Clinical risk was assessed using the European System for Cardiac Operative Risk Evaluation (EuroSCORE) (15). EuroSCORE is a combined measure of procedural mortality risk based on 17 factors comprising patient-related factors (eg, age, sex), cardiac-related factors (eg, unstable angina, recent myocardial infarction), and surgery-related factors (eg, surgery on thoracic aorta). Items were scored in accordance with the “logistic EuroSCORE” method to generate a percentage mortality risk estimate; further details of the scoring method can be found on the EuroSCORE web site (www.euroscore.org/logisticEuroSCORE.htm). In addition, we recorded whether a patient underwent cardiopulmonary bypass. Participants were asked to report any long-standing illnesses apart from heart disease before surgery (eg, cancer, thyroid disorder, diabetes); responses were summed to compute a chronic illness burden variable.

### Statistical analyses

A composite outcome was created combining MACE and mortality. Cox proportional hazards models were used to determine relationships between cortisol before surgery and clinical outcome; when a patient experienced more than one MACE, the earliest time interval from baseline was analyzed. Separate models were fitted for the cortisol slope over the day, cortisol AUC, and waking and evening values.

Because of the low number of clinical events (n = 18), only three covariates were included in the Cox regression models to avoid overfitting. Therefore, we included those covariates deemed most clinically relevant: EuroSCORE, whether the patient underwent cardiopulmonary bypass, and chronic illness burden. Because depression is known to be an important predictor of mortality in patients with CVD (16) and is also known to affect HPA axis activity (17), we examined associations between presurgical depression scores and presurgical cortisol parameters. There were no significant associations between depression and cortisol (all *P* > .05). Therefore, we did not include pre-surgical depression as a covariate in the analyses. Age and sex were not adjusted for separately in the Cox regression models because both age and sex are included in the EuroSCORE.

Associations between presurgical cortisol and covariates were examined using Pearson's correlations for continuous data and independent *t* tests for categorical variables. The significance level was set to *P* < .05 for all analyses, with precise *P* values reported for all test results. All statistical analyses were performed using SPSS version 20.0 (SPSS Inc).

## Results

Table 1 summarizes the characteristics of the patients. The sample had an age range of 44–90 years, was predominantly male (86.4%), and was overweight (body mass index [BMI] > 25 kg/m^2^, 81.6%). Just under one-fourth of the patients were diabetic (24%). The majority had on-pump cardiopulmonary bypass surgery (79.2%). In the years after surgery (mean = 2.68 y; SD = 0.40), nine patients (3.6%) experienced a MACE and 10 patients (4.0%) died, with one individual experiencing both outcomes. Cortisol slope, cortisol AUC, and waking cortisol levels were not significantly associated with EuroSCORE, cardiopulmonary bypass, or chronic illness burden. Evening cortisol levels were associated with EuroSCORE (*r* = 0.14; *P* = .030) but not with cardiopulmonary bypass or chronic illness burden. Occurrence of death or MACE after surgery was associated with EuroSCORE (*r* = 0.24; *P* < .001) and chronic illness burden (*r* = 0.19; *P* = .003).

**Table 1.** Demographic, Cortisol, and Clinical Characteristics of the Sample at Baseline and Follow-Up (n = 256)

Characteristic	
Age, y	68.10 ± 8.87
Female	34 (13.6)
BMI, kg/m^2^	28.84 ± 4.37
Smoker	20 (8.0)
Comorbidities	
Diabetes	60 (24.0)
Chronic illness burden	
No other chronic illness	156 (62.4)
1 other chronic illness	74 (29.6)
2 other chronic illnesses	20 (8.0)
Presurgical measures of cortisol	
Slope, nmol/L/h	1.67 ± 1.31
AUC, nmol/L	8874.55 ± 2770.55
Waking cortisol, nmol/L	19.45 ± 8.71
Time of waking, h:min	06:56 ± 01:12
Average evening cortisol, nmol/L	4.37 ± 3.81
Clinical factors	
Logistic EuroSCORE, %	4.49 ± 3.06
No. of grafts	2.97 ± 1.13
On-pump	198 (79.2)
Long-term recovery	
MACE	9 (3.6)
Deceased	10 (4.0)

Data are expressed as mean ± SD or number (percentage), unless stated otherwise.

### Presurgical cortisol and clinical outcomes

**Figure 1.**
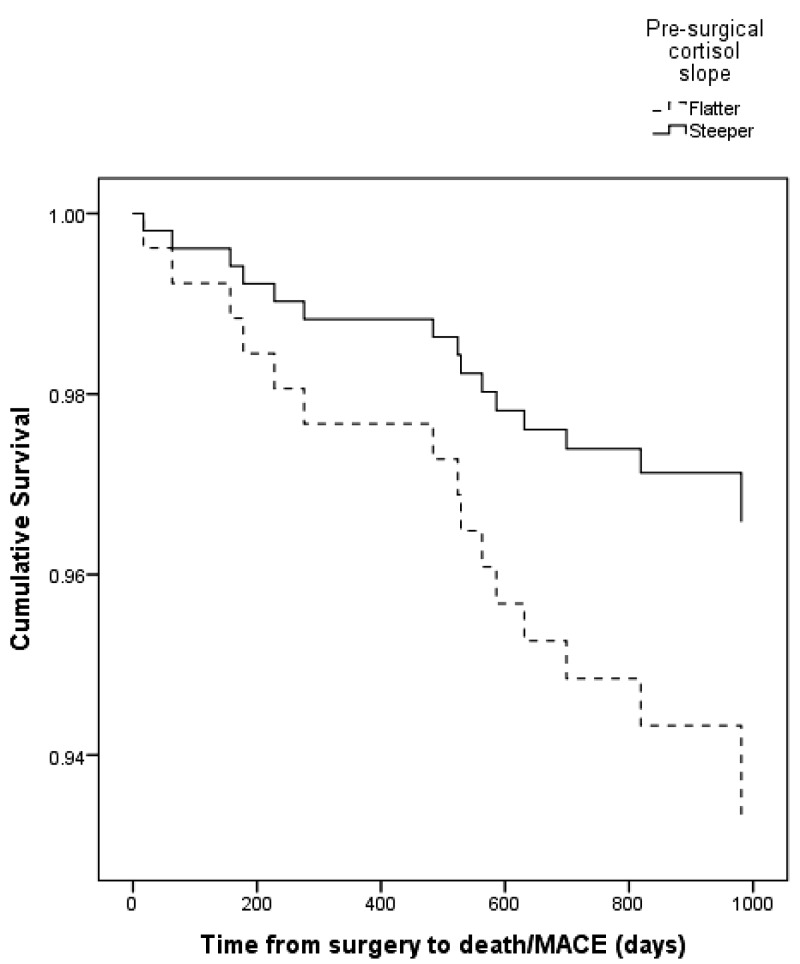
Kaplan-Meier survival curves for patients split into two equal groups at the median diurnal cortisol slope. This median split was performed only for illustrative purposes.

Diurnal cortisol slope predicted the occurrence of death or MACE after CABG surgery (hazard ratio = 0.73; 95% confidence interval [CI] = 0.56–0.96; *P* = .023). Patients with a steeper cortisol decline over the day were at reduced risk of experiencing adverse clinical outcomes (Table 2). More specifically, these results indicate that for every 1 nmol/L/h increase in cortisol slope, the risk of death or MACE fell by 27%. Chronic illness burden (*P* = .035) and EuroSCORE (*P* = .002) also predicted death or MACE after surgery. These analyses were repeated after excluding immediate events (three events) that occurred in the 5-day postoperative period. A steeper presurgical cortisol slope remained predictive of reduced risk of adverse clinical outcomes (HR = 0.70; 95% CI = 0.52–0.94; *P* = .017). For every 1 nmol/L/h increase in cortisol slope, the risk of death or MACE after the 5-day postoperative period fell by 30%. These survival analyses were carried out treating cortisol slope as a continuous variable, but for descriptive purposes participants were split into two equal groups based on cortisol slope using a median split. Cortisol changes over the day ≤ 1.68 nmol/L/h were considered indicative of “flatter” slopes. Kaplan-Meier survival plots of the two groups are shown in Figure 1. This plot reveals that divergence in survival/occurrence of MACE as a function of cortisol slope emerges very soon after CABG surgery.

**Table 2.** Results of Cox Regression Analysis, Showing Predictive Effects of Cortisol Slope and Covariates on the Occurrence of MACE and/or Death in the Years After CABG Surgery

Variable	Coefficient (B)	SE	Wald χ^2^	*P*	Hazard Ratio	95% CI
Cortisol slope	−0.31	0.14	5.16	**.023**	0.73	0.56–0.96
Chronic illness burden	0.67	0.32	4.46	**.035**	1.96	1.05–3.65
EuroSCORE	0.18	0.06	9.27	**.002**	1.20	1.07–1.35
Bypass^a^	−0.28	0.67	0.18	0.67	0.75	0.20–2.79

This model includes MACE/mortality cases that occurred within the 5-day postoperative period. Bold face indicates *P* values with statistical significance (*P* > .05).

a Whether the patient underwent cardiopulmonary bypass.

A flatter cortisol slope across the day can be due to low cortisol output on waking and/or higher evening cortisol values. We therefore examined associations between both waking and evening cortisol and clinical outcome. Waking cortisol was inversely associated with clinical outcome (HR = 0.93; 95% CI = 0.88–0.98; *P* = .011), suggesting that higher cortisol output on waking is linked to event-free survival. Evening cortisol levels were also significantly associated with clinical outcome (HR = 1.09; 95% CI = 1.014–1.170; *P* = .019), indicating that higher evening cortisol is linked to MACE or death in the years after surgery.

We also examined the association between cortisol AUC and adverse clinical outcomes. Presurgical AUC did not predict survival or the occurrence of MACE in the years after bypass surgery (*P* = .271). Excluding death or MACE that occurred in the 5-day postoperative period did not change these results.

## Discussion

The results of the study suggest that a flatter diurnal cortisol slope before surgery predicts the occurrence of MACE or mortality in CABG patients. Cortisol was sampled 1 month before surgery, so it does not reflect acute anticipatory stress responses before surgery. Our findings suggest that a flatter cortisol slope is related to poorer long-term outcomes in a patient sample with advanced CVD after bypass surgery and that this association is being driven by alterations in both waking and evening cortisol. These associations were independent of EuroSCORE, whether or not the patient underwent cardiopulmonary bypass, and chronic illness burden. There was no association between presurgical cortisol AUC and adverse clinical outcomes in the years following surgery.

To our knowledge, this is the first study to examine the association between diurnal cortisol and the occurrence of MACE or mortality in the years after CABG surgery. Our findings are in line with research that has reported associations between flatter diurnal slopes and adverse clinical events in other serious illnesses. For example, a flatter cortisol slope has been found to predict worse prognosis and mortality in metastatic breast cancer, lung cancer, and epithelial ovarian cancer (18–20). Flatter diurnal rhythms have also been associated with psychological stress and adversity, including depression (21), early childhood adversity (22), chronic stress (23), aging, and visceral obesity (1). Many of these factors are associated with coronary heart disease incidence (24) and also contribute to recurrent events and mortality in patients with advanced disease (25). Patients with heart disease have been found to have a flatter cortisol rhythm compared with healthy controls (26). The association between cortisol rhythm and mortality and adverse events in the current sample indicates that HPA axis dysregulation may increase with disease progression.

Our results indicate that both waking and evening cortisol levels predicted adverse outcomes for CABG patients, so the adverse effects of flatter profiles are not the result only of reduced waking concentration or elevated evening values. Kumari et al (12) found that an association between flatter cortisol slope and CVD mortality in a nonclinical sample was driven primarily by changes in evening levels of cortisol only. One reason for this discrepancy may be that HPA axis dysregulation has progressed further in individuals with advanced CVD. Fatigue and vital exhaustion are associated with cortisol output, as well as being risk factors for the occurrence of adverse cardiac events over time (27, 28). Associations between lower levels of waking cortisol and fatigue have been reported in older adults and coronary artery disease patients (29, 30). It is possible that fatigue or exhaustion may be a factor influencing the association between lower waking cortisol and adverse outcomes in the current study.

It is likely that one of the factors contributing to the link between cortisol rhythm and MACE/mortality is inflammation. The role of inflammation in atherosclerosis is well established, with higher levels of proinflammatory cytokines being associated with enhanced plaque instability and acute thrombotic complications (31, 32). Elevations of inflammatory markers are also independent predictors of future cardiovascular events (33). The HPA axis is one of the major mechanisms by which inflammation is regulated. Cortisol exerts an immunomodulatory effect inhibiting the expression of inflammatory cytokines such as IL-6, IL-1, and TNF-α (34). Dysregulation of the HPA axis may result in the impairment of inhibition, leading to sustained high levels of inflammation (35). Nijm et al (26) found that individuals with coronary artery disease had a flattened cortisol slope compared with healthy controls and that levels of evening cortisol were strongly correlated with serum levels of IL-6 and C-reactive protein. Similarly, a flattened cortisol slope has been associated with higher circulating levels of plasma IL-6 in epithelial ovarian cancer (20) and metastatic colorectal cancer (36).

CABG surgery leads to substantial increases in cortisol concentration that decline over the postoperative period and is coupled with alterations in sensitivity to ACTH (37). Mechanistically, dysregulation of the HPA axis is likely caused in part by diminished sensitivity of the main corticosteroid receptors—the glucocorticoid receptor (GR) and the mineralocorticoid receptor (MR). Cortisol exerts its effects by binding to the GR and MR, which subsequently down-regulates proinflammatory gene transcription (38). With reduced corticosteroid receptor sensitivity, cortisol is no longer able to exert its regulatory effects successfully, leading to a breakdown in the HPA axis negative feedback loop and an increase in the intensity of the inflammatory response (39). GRs are expressed in cardiovascular tissue and are responsible for maintaining vascular tone and modifying vascular inflammatory responses (40). GRs have also been shown to exert anti-inflammatory actions in cardiac muscle cells (41). MRs are expressed in vascular smooth muscle cells and play a role in regulating vascular function (42). Moreover, spironolactone, a MR antagonist, is a powerful antifibrotic used in the treatment of heart failure and atrial fibrillation (43). Therefore diminished sensitivity of the corticosteroid receptors may have direct effects on the heart. Diminished GR sensitivity has been associated with a flatter diurnal cortisol slope (44). It is possible that the association between diurnal cortisol slope and MACE or mortality observed in the current study reflects reduced sensitivity of the corticosteroid receptors. Future research should assess both diurnal cortisol rhythm and GR and MR function in CVD patients.

In our study, we found no association between overall cortisol output (AUC) and MACE or mortality after CABG surgery. A study of lung cancer survival also found that cortisol AUC had no predictive value in terms of mortality (18). This adds support to the notion that measuring cortisol slope across the day is likely to be a more useful prognostic tool than total cortisol output and that the diurnal rhythm may be related to specific circadian mechanisms of inflammation (45).

A strength of this study is that cortisol was measured repeatedly across a day several weeks before surgery. The pattern of output may therefore represent habitual profiles rather than being affected by acute anticipation of hospitalization. The study had a prospective design, and attrition was low, with ascertainment of clinical outcomes in more than 95% of participants. However, the sample size was relatively small, with MACE and death occurring in only 18 participants. The ARCS study was not specifically designed to investigate cortisol and cardiac outcomes, and this limited statistical power and reduced the number of covariates that could be included in the analyses. Larger studies of patients with advanced cardiac disease are needed to establish the robustness of the findings. Our sample was largely composed of white men of European origin, so the results may not be readily generalizable to other groups. We were unable to access information about specific causes of death for all patients who died. Cortisol was measured over a single day, meaning that the diurnal rhythm may have been affected by situational rather than chronic factors. Nighttime cortisol was not measured, so it was not possible to assess total 24-hour cortisol exposure. Furthermore, these data do not prove a causal connection between diurnal cortisol slope and MACE or mortality in these patients; although we included important clinical covariates, there might be unmeasured factors influencing diurnal cortisol rhythms that also increased risk of adverse outcomes.

In conclusion, our results indicate that a flatter diurnal cortisol slope before surgery is associated with poorer long-term outcomes in patients undergoing coronary revascularization. Diurnal cortisol profiles can be obtained without difficulty because the measures are noninvasive and samples are stable for several days. Measuring diurnal cortisol rhythm may help to identify patients at risk of adverse events or death, allowing additional support and care to be provided.
